# Hyaluronan/Poly-L-lysine/Berberine Nanogels for Impaired Wound Healing

**DOI:** 10.3390/pharmaceutics13010034

**Published:** 2020-12-28

**Authors:** Giovanni Amato, Maria Aurora Grimaudo, Carmen Alvarez-Lorenzo, Angel Concheiro, Claudia Carbone, Angela Bonaccorso, Giovanni Puglisi, Teresa Musumeci

**Affiliations:** 1 Department of Drug Sciences, University of Catania; V.le Andrea Doria, 6, 95125 Catania, Italy; ccarbone@unict.it (C.C.); angela.bonaccorso@unict.it (A.B.); puglisig@unict.it (G.P.); teresa.musumeci@unict.it (T.M.); 2 Departamento de Farmacología, Farmacia y Tecnología Farmacéutica, I+D Farma (GI-1645), Facultad de Farmacia and Health Research Institute of Santiago de Compostela (IDIS), Universidade de Santiago de Compostela, 15782 Santiago de Compostela, Spain; carmen.alvarez.lorenzo@usc.es (C.A.-L.); angel.concheiro@usc.es (A.C.)

**Keywords:** hyaluronan, ε-polylysine, berberine, chronic wounds, nanogels

## Abstract

Physiological wound healing process can be delayed in the presence of certain pathologies, such as diabetes or cancer. In this perspective, the aim of this study was to design a new nanogel platform of hyaluronan, poly-L-lysine and berberine suitable for wound treatment. Two different nanogel formulations were selected after a first formulation screening. They were prepared by adding dropwise 2 mg/mL hyaluronan aqueous solution (200 or 700 kDa) to 1.25 mg/mL poly-L-lysine aqueous solution. Blank nanogels formulated with 200 kDa HA resulted stable after freeze-drying with dimensions, polydispersity index and zeta potential of 263.6 ± 13.1 nm, 0.323 ± 0.029 and 32.7 ± 3.5 mV, respectively. Both blank and berberine-loaded nanogels showed rounded-shape structures. Loaded nanogels released nearly 50% of loaded berberine within 45 min, whereas the remaining 50% was released up to 24 h in vitro. Both, blank and berberine-loaded nanogels were able to completely close the fibroblasts gap in 42 h.

## 1. Introduction

Wound healing is described as a complex biological process aimed at inducing wound repair and preventing exaggerated or delayed responses [1,2]. However, chronic wounds can fail to follow this physiologically ordered sequence of events, entering a cycle of inflammation and bacterial infection [3]. Also, wound chronicity was demonstrated often associated with age-related pathologies, diabetes, vascular insufficiency or cancer [4].

High levels of exudate promote bacterial-growth-producing endotoxins and pro-inflammatory toxins, leading to wound inflammation [5,6]. *Staphylococcus*, *Pseudomonas*, *Corynebacterium* and *Anaerococcus* species are among the most abundant bacteria present in chronic wounds [7]. As a consequence of bacterial contamination, chronic wounds may be arrested in an inflammatory state associated with a high amount of cytotoxic enzymes, oxygen-free radicals and inflammatory leucocytes [8]. Wound debridement and topical application of antimicrobials still represent conventional therapy for chronic wounds [3]. Among all, wound treatment with local therapies presents advantages such as localized antibacterial release and reduced drug dose, adverse effects and antibiotic resistance [7]. In this regard, innovative nanocarriers can be designed as optimized drug delivery systems to avoid microbial contamination at the wound site and disrupt the biofilms [3].

Hydrogel nanoparticles (or nanogels) are defined as nanometer-sized hydrogels presenting three-dimensional networks of crosslinked polymer chains [9]. These nanocarriers show high water content and swelling properties typical of hydrogels, and very small dimensions with a large surface area like nanoparticles [10,11]. Thanks to their structure, nanohydrogels can incorporate a high volume of water, acting as moisturizing agents and enhancing stratum corneum hydration [12], which favors their role in the skin drug delivery field. However, few limitations associated with their use have to be mentioned, such as low drug encapsulation efficiency and poor control of drug release [13].

Hyaluronic acid (HA) is a linear anionic polysaccharide consisting of a repeating disaccharide of (1-4)-linked β-d-glucuronic acid and (1-3) N-acetyl β-d-glucosamine monomer. The viscoelasticity, biocompatibility and non-immunogenicity properties of this natural polymer have been reported to facilitate the healing and regeneration of surgical wounds [14]. HA can be thus employed in burns, surgical and chronic wounds providing a temporary structure, facilitating nutrients diffusion, ridding the wound of metabolic waste products by interaction with receptor CD44 and controlling hydration. Moreover, this polysaccharide is thought to be involved in keratinocyte migration and proliferation [15], and low MW HA fragments (100 to 300 kDa) promote wound repair and increase the self-defense of skin epithelium [16]. HA also regulates skin cell proliferation by interacting with CD44 receptors [17].

Antimicrobial peptides, essential natural components of innate immunity against pathogenic organisms, have recently attracted much attention as a novel way for fighting infections [18]. Among them, ε-polylysine (PL) is a cationic homopolyamide naturally produced by the filamentous bacterium *Streptomyces albulus*, water-soluble, biodegradable and non-toxic, formed by a hydrophilic cationic linear homopoly(amino acid) of 25–35 L-lysine residues [19]. This peptide shows antibacterial activity against a wide spectrum of pathogens, including both Gram-positive and Gram-negative bacteria [20,21,22] and fungi [23]. Indeed, the electrostatic adsorption of PL to the bacteria surface seems responsible for lipopolysaccharide stripping and permeabilization of the outer membrane, leading to bacteria cell damage [23]. The surface rich in cationic amino groups is responsible for antibacterial activity and enhancing cellular drug uptake and transport. Once entered into the cytoplasm, PL can also induce the generation of ROS response, alteration of various gene expressions and several repair mechanisms, which can contribute to cell death [24]. For this reason, this polymer has been already used as a natural antimicrobial agent to preserve packaged food [25].

PL may interact with anionic polymers by electrostatic interactions, and it has been widely used to obtain polyelectrolyte multilayers by the sequential deposition of the oppositely charged polymers onto a solid surface [26]. The layer-by-layer assembly of HA and PL has been demonstrated to be a promising approach for creating biomaterials able to mimic the native extracellular matrix and control drug release [27,28,29]. For example, biomimetic multilayer films of HA and PL have been designed to mimic the extracellular matrix of neural tissues, obtaining an enhancement of the neurite outgrowth, regulation of neuron differentiation and the formation of an organized network [27]. Multilayers of PL and HA on top of gold nanostructures demonstrated a superior differentiation of human adipose-derived stem cells into chondrogenic and osteogenic lineages in comparison to adipogenic lineages [28]. Also, a tumor microenvironment-responsive polymer carrier obtained layer-by-layer has been shown able to actively target tumors exploiting the interactions of hyaluronic acid with CD44 receptors [29].

Apart from layer-by-layer structures, PL is expected to interact with HA forming inter- and intra- molecular linkages between PL amino groups and HA carboxylic groups, which may generate macro-, micro- or nanohydrogels [30,31]. It can be hypothesized that the dual presence of HA and PLL could be very useful for promoting tissue regeneration while offering protection against bacterial contamination.

The correlation between low antioxidant levels and impaired wound healing suggests a possible use of antioxidants in chronic wound management [32] as an excessive ROS concentration may lead to oxidative stress, which further impairs and slows down the wound healing process [33]. Berberine shows a wide range of pharmacological activities, such as antioxidant, antibacterial, antifungal and anti-inflammatory [34]. This antioxidant is also reported to increase the activity of antioxidant enzymes such as catalase or glutathione peroxidase [34], and it suppresses oxidative stress and inflammation through multiple mechanisms [35]. Besides antioxidant properties, the anti-inflammatory activity of berberine is also associated with the inhibition effect on the mitogen-activated protein kinase signaling pathways [35].

**Figure 1:**
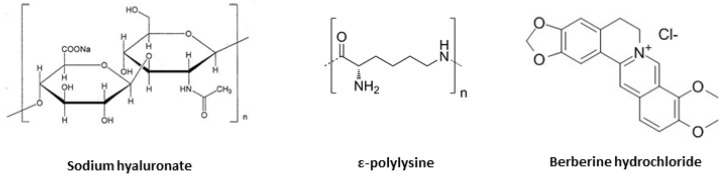
Chemical structures of sodium hyaluronan (HA), ε-polylysine (PL) and berberine hydrochloride.

The aim of the present work was to combine the abovementioned features of HA, PL and berberine (Figure 1) into nanogels suitable for wound treatment. A simple ionotropic gelation process was implemented to produce nanogels. Preliminary formulation design was performed by varying HA MW and the molar ratio between HA and PL. Selected formulations were studied in terms of particle size, zeta potential, morphology, stability after freeze-drying, release pattern and capability for wound closure in vitro.

## 2. Materials and Methods

### 2.1. Materials

Sodium hyaluronate (HA, MW 200, 700 or 1200 kDa, Figure 1) was kindly supplied by Fidia Farmaceutici S.p.a. (Noto, Italy). Sterile water was used for all the preparations (0.2 µm Polyethersulfone membranes, Puradisc 25 AS, Whatman™, Maidstone, UK). Epsiliseen^®^-H (ɛPL, ɛpolylysine, MW 4.7 kDa, Food Grade, Figure 1) was purchased from Siveele B.V. (Breda, The Netherlands). Potassium hydroxide (KOH, pellets, 85%) was from Carlo Erba Reagents S.A.S. (Barcelona, Spain), while hydrogen chloride (HCl, reagent grade, 37%) was purchased from Sigma Aldrich (St. Louis, MO, USA). Berberine hydrochloride was supplied by Farmalabor Srl (Canosa di Puglia, Italy, Figure 1). Spectra/Por^®^ Dialysis Membranes (MWCO 3.5 kDa) were purchased from Repligen Corporation (Waltham, MA, USA). NaCl was from Panreac Quimica S.L.U. (Barcelona, Spain).

### 2.2. Preliminary Design of Nanogels

In total, 1 mL of HA aqueous solution (2 or 5 mg/mL) was added dropwise to 0.5, 0.75, 1.0 or 1.25 mg/mL ɛPL aqueous solution (1 or 2 mL) at room temperature under magnetic stirring (700 rpm) (formulations listed in Table 1). Before mixing, HA and ɛPL solutions were adjusted to pH~7 and pH~6 using 5 µL of KOH 50 mM and HCl 1.2 M, respectively. After magnetic stirring for 1 or 2 h, formulations were centrifuged (12,000 rpm, 1 h, 4 °C) (ThermoScientific™ SL16R, ThermoFisher Scientific, Waltham, MA, USA) for collecting nanogels at the bottom. Supernatants were discarded and pellets resuspended in sterile water.

### 2.3. Light Scattering

Particle size (detection angle of 90°, room temperature) and zeta potential (ZP) measurements were performed using a Zetasizer Nano S90 (Malvern Instruments, Malvern, UK). Analyses were performed in triplicate for each batch.

### 2.4. Stability after Freeze-Drying

Nanogel formulations were frozen at −20 °C overnight and then freeze-dried (Modulyo^®^ Freeze Dryer, Thermo Electron Corporation, Waltham, MA, USA) obtaining a dried powder. Reconstitution was performed by adding sterile water (the same volume as initially in the vial). Reconstituted formulations were analyzed in terms of size and ZP (see Section 2.3). Process yields were calculated by multiplying by one hundred the ratio between the mg of the obtained freeze-dried product and the initial mg of used components during the preparation. Experiments were carried out in triplicate.

### 2.5. Selected Nanogels Preparation and Berberine Loading

In total, 2 mg/mL HA (200 or 700 kDa) aqueous solution was added dropwise to 1.25 mg/mL ɛPL aqueous solution (1:1 voL/voL) at room temperature under magnetic stirring (700 rpm). Before mixing, HA and ɛPL solutions were adjusted to pH~7 and pH~6 using 5 µL of KOH 50 mM and HCl 1.2 M, respectively. After magnetic stirring, formulations were centrifuged (12,000 rpm, 1 h, 4 °C, ThermoScientific™ SL16R, ThermoFisher Scientific, Waltham, MA, USA) for collecting nanogels at the bottom. Pellets were finally resuspended in sterile water. For preparing drug-loaded formulations (NG6L, NG7L), berberine hydrochloride was dispersed in HA solution (200 kDa, 2 mg/mL) in a concentration equal to 1.5 mg/mL under magnetic stirring and then the solution added dropwise to 1.25 mg/mL ɛPL solution, as previously described.

Particle size and ZP values were investigated by light scattering (see Section 2.3). Experiments were carried out in triplicate. The percentage of drug loading was calculated by dividing the mg of berberine by the mg of lyophilized nanogels and multiplied by 100.

### 2.6. Nanogels Morphology

Morphological analysis of selected nanogels was performed by using a high-resolution transmission electron microscope (JEM-1011, JEOL USA Inc., Peabody, MA, USA). Briefly, nanogel formulations were placed on carbon-coated grids and 5 μL of 2% *w*/*v* phosphotungstic acid was added for 60 s. After washing with pure water, the excess was carefully removed and samples dried before observation.

### 2.7. Berberine Release Studies

Dialysis bags (Spectra/Por^®^ membranes, MWCO 3.5 kDa) were filled with 1 mL of berberine loaded nanogel formulation (NG6L) and immersed in 12 mL NaCl 0.9% *w*/*v*. The systems were maintained under magnetic stirring (500 rpm) at 37 °C up to 24 h. Release medium (1 mL) was sampled at different time points and fresh saline solution (1 mL) was added to the system. Collected samples were analyzed using UH5300 UV-Visible Spectrophotometer (Hitachi, Chiyoda, Japan) at 345 nm and calibration curve built in the range 5–25 µg/mL (R^2^ = 0.9994). Blank nanogel formulation (NG6B) was used as control. Release studies were performed in triplicate using the same formulation batch.

### 2.8. In Vitro Fibroblasts Migration Assay

Blank and berberine loaded nanogels (NG6B and NG6L) were investigated to assess their wound closure properties against murine fibroblasts (CCL-3T3, ATCC, LGC Group, London, UK). Cells seeding was performed in μ-dishes (Ibidi, Giardini, Italy) composed of two chambers (1.5 × 10^4^ cells/chambers) divided by a 500 μm cell-free gap. Fibroblasts were cultured in DMEM medium (Dulbecco′s modified Eagle′s Medium, 10% *v*/*v* fetal bovine serum, 1% *v*/*v* penicillin-streptomycin). After 24 h culture at 37 °C/5% CO_2_, the inner insert was removed, displaying two cell leaflets divided by a gap with prefixed dimensions. Nanogels were prepared by resuspension in DMEM medium after the centrifugation step (see preparation method in Section 2.5) and diluted in DMEM medium (dilution factor was equal to 10). One milliliter was placed in contact with cells for 48 h at 37 °C/5% CO_2_. Control included cells with no contact with formulations. The μ-dishes were then incubated at 37 °C, in 5% CO_2_, and 95% relative humidity. Cell migration in the gap area was evaluated by using a Nikon Eclipse TS100 Microscope (Chiyoda, Japan) and a microscope digital camera system Olympus DP12 (Shinjuku, Japan) at different time points. The percentages of cell gap area were calculated with regard to the t_0_ using ImageJ software (NIH, Bethesda, MD, USA).

### 2.9. Statistical Analysis

Data were analyzed using ANOVA and multiple range tests (Statgraphics Centurion XVI 1.15, StatPoint Technologies Inc., Warrenton VA, USA). Differences were considered statistically significant when *p* < 0.05. In the text, all data are reported as mean value ± sd.

## 3. Results and Discussion

### 3.1. Nanogels Formulative Studies

Generally, polymeric nanogels are broadly classified according to their crosslinked structure into chemically crosslinked nanogels involving covalent bonds and physically crosslinked nanogels involving self-assembling through weaker linkages by non-covalent bonds. Among them, the main difference is the presence of a stable and rigid polymeric network in case of chemical crosslinking, while physical interactions lead to a weaker polymer chain entanglement [36,37]. Nevertheless, physically crosslinked nanogels offer the advantage of mild preparation conditions in aqueous solution following the green chemistry principles [9]. Indeed, this new branch of chemistry with ecological approaches is aimed at reducing or eliminating the use of harmful substances in chemical processes as well as dropping harmful and toxic products [38]. For all the above-mentioned reasons, we decided to design a novel physically crosslinked nanogel platform to avoid the use of any toxic reagent and offer good translational properties thanks to its simplicity.

Generally, nanogels physical characteristics such as dimensions are reported to be affected by the preparation conditions, the concentration of used polysaccharide/polymer in the formulation, physicochemical characteristics of all the compounds used in the formulation, while crosslinker characteristics and concentration impact the polydispersity and zeta potential values [39].

Firstly, we decided to add a small volume of basic and acid solutions to HA and PL solutions prior to mixing, respectively, in order to maximize electrostatic interactions between the two components during nanogels preparation [40]. Indeed, the pKa of HA in solution is reported to be 3.0 and that of PL about 10 [29]. Moreover, it is reported that PL presents secondary structures that vary with pH in aqueous solution, adopting a random coil conformation under acidic and neutral conditions, and undergoing a coil-to helix transition under basic conditions [41]. Thus, we assumed that PL conformation at pH 6 is essential for maximizing PL interactions with HA.

Then, the proper time for nanogel formation was studied. In total, 1 mL of 2 mg/mL 200 kDa-HA solution was added dropwise to 1 mL of 0.5 mg/mL PL solution, and the influence of magnetic stirring duration was evaluated by comparing dimensions and PDI obtained maintaining the formulation under magnetic stirring for 1 or 2 h. Lower PDI values were obtained after one-hour magnetic stirring in comparison to the longer magnetic stirring, while dimensions were comparable (data not shown). Thus, a 60 min time was selected as the time for magnetic stirring during nanogel preparation.

After, different nanogel compositions were tested, and the results in terms of particle size, PDI and ZP after post-centrifugation resuspension are shown in Table 1. PDI results were used as discriminating parameters for the selection of nanogel formulations. Observing the behavior of 200 kDa-HA 2 mg/mL solution, when the ratio of the number of moles of repetitive units of ε-PL to the ones of HA was equal to 3, ZP values resulted close to 0, leading to nanogel aggregation, for this reason, this ratio was not tested for the other molecular weights. For 5 mg/mL 200 kDa-HA solution, the optimum molar ratio was found to be equal to 1.2. Using a 1.5 mole ratio, higher PDI values were observed. Dimensions and PDI values obtained with 2 and 5 mg/mL 700 kDa-HA solutions agreed with the data obtained by using 200 kDa-HA, while 1200 kDa-HA based nanogels were obtained only by using a 2 mg/mL polymer solution, probably due to the high viscosity of 5 mg/mL 1200 kDa-HA aqueous dispersions.

The five most promising nanogel formulations (codes A, C, E, F and H in Table 1) were selected for further characterization, that is, the evaluation of process yields and stability after freeze-drying. Process yields were low for all formulations (approximately 10% in all cases) and nanogels were found to aggregate after freeze-drying (data not shown). These preliminary results demonstrated that nanogel formulations needed a further optimization process for obtaining higher yields and stable nanogels after freeze-drying.

Firstly, 700 kDa-HA solution (2 mg/mL) was added dropwise to 0.75, 1 or 1.25 mg/mL ε-PL solution; PDI value lower than 0.3 was obtained only using 1.25 mg/mL ε-PL solution. Conversely to the previously obtained results, positive ZP values were obtained in the case of nanogels with 1 or 1.25 mg/mL ε-PL concentration, suggesting that the amount of PL was sufficient for efficient nanogels formation. Moreover, positively charged nanoparticles generally show a good capability to interact with the negative charges present on the skin surface [42]. For these reasons, the highest PL concentration (1.25 mg/mL) was selected for further investigation. The occurrence of macrogelation was observed in the case of 2 mg/mL 1200 kDa-HA solution, probably due to the high MW of the polymer impairing the formation of monodisperse nanogels (data not shown). In the case of 5 mg/mL HA (200 or 700 kDa) solution, high values of PDI (>0.3) were found and the formulations were discarded from further investigation (data not shown).

From the gathered information, two different nanogel formulations were selected, obtained by adding dropwise 2 mg/mL HA aqueous solution (200 or 700 kDa, coded as NG6 and NG7, respectively) to 1.25 mg/mL ε-PL aqueous solution. Nanogel characteristics (blank and loaded formulations) are listed in Table 2. The percentages of drug loading resulted very low (approx. 5%) for both loaded nanogel formulations, but in line with the reported literature [43]. As shown in Table 2, berberine loading slightly increased nanogel particle size, while no different results were obtained in terms of PDI or ZP values.

Blank nanogels formulations were freeze-dried and then light scattering analysis was performed. Blank nanogels formulated with 700 kDa-HA showed PDI values superior than 0.7 after freeze-drying and thus discarded for further investigation, while blank nanogels formulated with 200 kDa-HA resulted as stable after freeze-drying. Dimensions, PDI and ZP values post the freeze-drying of blank NG6 were 263.6 ± 13.1, 0.323 ± 0.029 and 32.7 ± 3.5 mV, respectively.

### 3.2. Nanogels Characterization

NG6B and NG6L were further investigated in terms of stability under storage, release pattern, morphology and in vitro capability of promoting cell migration.

**Figure 2:**
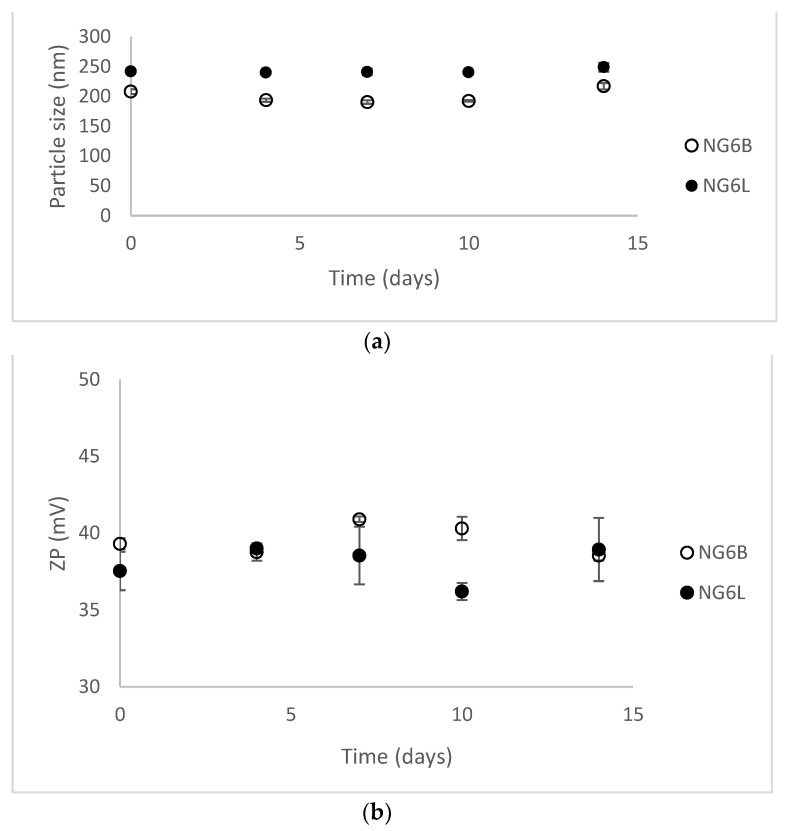
Stability results at 4 °C for 14 days in terms of dimensions (**a**) and ZP values (**b**) (mean ± sd, analysis in triplicate on single batch).

Nanogels were stored at 4 °C for a preliminary evaluation of short-term stability by monitoring dimensions and ZP values for two weeks (results shown in Figure 2). NG6B and NG6L resulted as stable (PDI values lower than 0.3 at each time point), presumably thanks to the highly positive ZP values of the formulations responsible for hindering nanogel aggregation in the solution.

**Figure 3:**
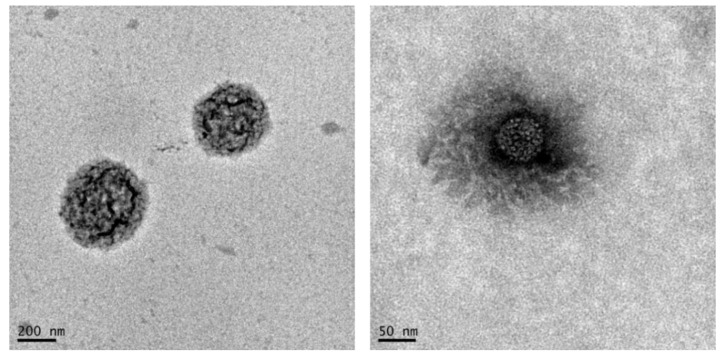
TEM images of blank (NG6B, on the **left**) and berberine loaded (NG6L, on the **right**) nanogels.

NG6B and NG6L were further investigated in terms of morphology by TEM (Figure 3). As shown, defined rounded-shape structures were detected in the case of NG6B, while a less defined structure was observed for NG6L. However, it has to be mentioned that the determination of the size of nanogels by TEM can be complicated by the fact that this property is highly dependent on the solvent the nanogels are dispersed in, and sample preparation required for TEM analysis may also lead to artifacts [44].

**Figure 4:**
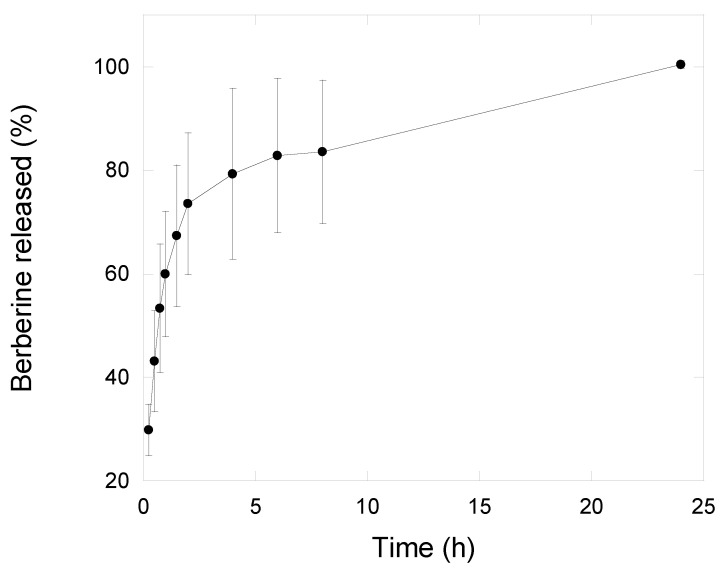
Berberine release profile from nanogels (NG6L formulation) recorded in saline solution using dialysis membranes setup (n = 3, mean ± sd).

NG6L formulation was further investigated in terms of berberine release using the dialysis bag method, a commonly used assay used to study drug release pattern from nanoparticles [45]. Generally, the release kinetics of nanogels show a diffusion predominant delivery mechanism [46], although the extent of swelling could have a strong impact on release pattern. Indeed, polymeric nanogels begin to swell after contact with biological fluids due to the relaxation of macromolecular chains and the drug diffusion out of the swollen polymer [47]. Nanogel swelling can thus promote the diffusion of the external phase into the nanogel bulk and the release of the drug [46]. As shown in Figure 4, nanogels released nearly 50% of the loaded drug within 45 min under sink conditions, whereas the remaining 50% was released in up to 24 h in vitro. A similar drug release pattern has been reported by other authors [13]. For the goal of this study, we hypothesize that a burst berberine release could be beneficial for assuring efficacious drug concentration in situ. Indeed, the concentration of berberine needed for 50% inhibition of lipid peroxidation, superoxide radical and nitric oxide radical is 100, 61 and 48 µg/mL, respectively [48], corresponding to approximately 3 mg of lyophilized loaded nanogels.

**Figure 5:**
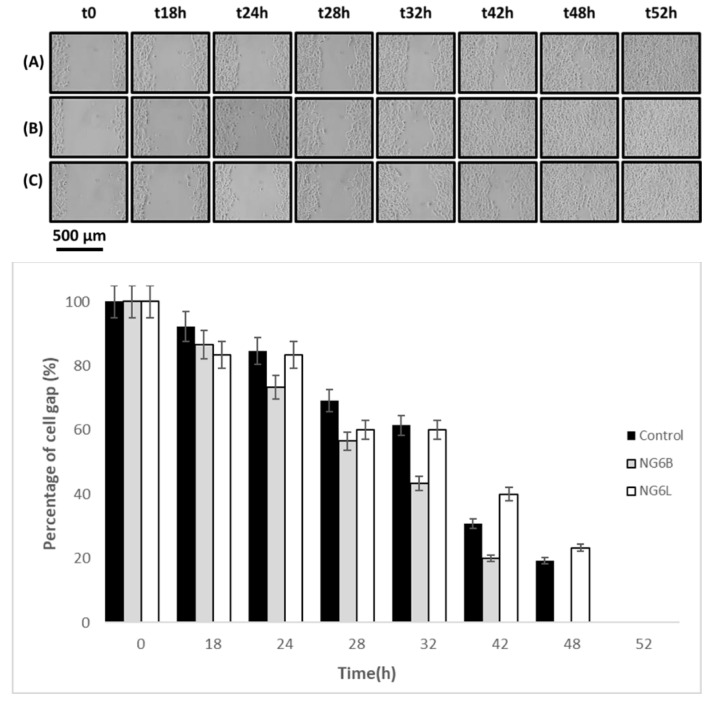
In vitro wound-healing assay photographs at 0, 18, 24, 28, 42, 48 and 52 h after incubation with the samples (control row A, NG6B row B, NG6L row C) at 37 °C in 5% CO_2_ and 95% relative humidity, and percentages of cell gap as a function of time.

Finally, the wound-closure-promoting properties of the developed nanogels (NG6B, NG6L) were studied using an in vitro wound-healing assay to preliminarily assess the potentiality of the formulated nanogels for wound-healing applications. As shown in Figure 5, the NG6B formulation wound closure rate was higher in comparison to the loaded formulation and control. Indeed, loaded nanogels were not able to completely close the cell gap, showing a comparable behavior to the control. This finding can be related to the electrostatic interactions occurring between HA and berberine, which made HA less available for promoting cell gap disclosure. Indeed, Zhao and coworkers demonstrated that berberine retarded wound healing in vitro of scratched breast cancer cells [49].

## 4. Conclusions

In this study, HA/PL nanogels were prepared by the ionotropic gelation method and their potential for wound healing was investigated. Promising nanogels were obtained by the simple mixing of two polymeric solutions, rendering this platform interesting for biomedical applications. After a preliminary study, we successfully developed monodisperse nanocarriers with 200 nm dimensions for wound healing applications and good stability after freeze-drying. TEM analysis confirmed the formation of nanostructured systems, while the berberine release study revealed that 50% of the loaded drug was released within 45 min. Depending on the application, the entrapment efficiency of the cargo should be improved and many strategies (such as forming complexes with cyclodextrins) are going to be considered.

## Figures and Tables

**Table 1:** Preliminary design of nanogels. Formulation composition and results in terms of size, PDI and ZP after crosslinking with ε-PL (n = 3, analysis in triplicate for single batch).

Code	ε-PL Solution		HA Solution			Molar Ratio n_ε-PL_/n_HA_	Post-Centrifugation		
	V (mL)	Conc (mg/mL)	V (mL)	Conc (mg/mL)	MW (kDa)		Size (nm)	PDI	ZP (mV)
**A**	**1**	**0.5**	**1**	**2**	**200**	**1.5**	**200.4 ± 4.4**	**0.161 ± 0.017**	**−38.8 ± 0.1**
B	2	0.5	1	2	200	3	505.7 ± 14.7	0.673 ± 0.056	−0.05 ± 0.14
**C**	**2**	**0.5**	**1**	**5**	**200**	**1.2**	**244.7 ± 4.0**	**0.089 ± 0.024**	**−38.8 ± 0.5**
D	2.5	0.5	1	5	200	1.5	254.7 ± 27.6	0.447 ± 0.060	−51.1 ± 2.6
**E**	**1**	**0.5**	**1**	**2**	**700**	**1.5**	**206.7 ± 8.5**	**0.207 ± 0.022**	**−40.9 ± 2.5**
**F**	**2**	**0.5**	**1**	**5**	**700**	**1.2**	**279.7 ± 9.4**	**0.256 ± 0.005**	**−55.4 ± 2.77**
G	2.5	0.5	1	5	700	1.5	243.6 ± 16.1	0.426 ± 0.046	1.18 ± 0.06
**H**	**1**	**0.5**	**1**	**2**	**1200**	**1.5**	**277.5 ± 5.7**	**0.273 ± 0.18**	**−44.2 ± 2.21**
I	2	0.5	1	5	1200	1.2	Macrogelation		
L	2.5	0.5	1	5	1200	1.5	Macrogelation		

**Table 2:** Characteristics of blank and loaded nanogels (mean ± sd, analysis in triplicate on 2 *, 3 ^#^, 4 ^§^ or 5 ^‡^ different batches).

Sample	Particle Size (nm)	PDI	ZP (mV)	Yield (%)	EE (%)
**NG6B** ^‡^	207.9 ± 7.3	0.232 ± 0.019	+40.9 ± 4.6	≈40	-
**NG6L** ^§^	245.2 ± 11.2	0.208 ± 0.050	+38.2 ± 1.6	≈50	≈10
**NG7B** ^#^	195.5 ± 9.9	0.169 ± 0.076	+36.0 ± 5.1	≈40	-
**NG7L** *	222.8 ± 19.4	0.136 ± 0.060	+37.7 ± 0.8	≈30	≈10

## Data Availability

The data presented in this study are available on request from the corresponding author. The data are not publicly available due to University intellectual property rights.
